# Supplemented Use of Pre-, Pro-, and Synbiotics in Severe Acute Pancreatitis: An Updated Systematic Review and Meta-Analysis of 13 Randomized Controlled Trials

**DOI:** 10.3389/fphar.2018.00690

**Published:** 2018-06-28

**Authors:** Xu Tian, Yuan-Ping Pi, Xiao-Ling Liu, Hui Chen, Wei-Qing Chen

**Affiliations:** ^1^Department of Gastroenterology, Chongqing Key Laboratory of Translational Research for Cancer Metastasis and Individualized Treatment, Chongqing University Cancer Hospital & Chongqing Cancer Hospital & Chongqing Cancer Institute, Chongqing, China; ^2^Editorial Office, TMR Integrative Nursing, TMR Publishing Group, Tianjin, China; ^3^Department of Nursing, Key Laboratory for Biorheological Science and Technology of Ministry of Education (Chongqing University), Chongqing University Cancer Hospital & Chongqing Cancer Hospital & Chongqing Cancer Institute, Chongqing, China

**Keywords:** severe acute pancreatitis, prebiotics, probiotics, synbiotics, enteral nutrition, meta-analysis

## Abstract

**Introduction:** The role of pre-, pro-, and synbiotics supplemented to standard enteral nutrition in severe acute pancreatitis (SAP) remains unclear. We performed this updated meta-analysis to determine the value of pre-, pro- and synbiotics supplemented to standard enteral nutrition in predicted SAP.

**Methods:** A systematic search in PubMed, EMBASE and Cochrane Central Register of Controlled Trials (CENTRAL) databases was performed. Eligible studies were randomized controlled trials (RCTs) that compared the effects of pre-, pro-, and synbiotics supplemented to standard enteral nutrition with control regime in predicted SAP patients. Risk ratio (RR) and mean difference (MD) with 95% confidence interval (95% CI) were used to express the estimates of dichotomous and continuous data respectively.

**Results:** 13 RCTs comprising an aggregate total of 950 patients were eventually enrolled. Pooled results suggested that supplemented use of pre-, pro- and synbiotics effectively shorten the length of hospital stay in Chinese SAP cohorts (6 RCTs, MD = −5.57, 95% *CI* = −8.21 to −2.93, *P* < 0.001); however significant differences with regard to remaining clinical outcomes were not detected for all patients. Further analysis based on category of interventions including pre-, pro- and synbiotics also confirmed the findings to be reliable.

**Conclusions:** Supplemented use of pre-, pro and synbiotics reduced the length of hospital stay in Chinese SAP cohorts. And thus, we concluded that pre-, pro- and synbiotics supplemented to standard enteral nutrition may be a potential option for the treatment of SAP patients. However, we also suggest designing further studies with large-scale and rigorous methods of addressing data to establish the effects and safety of supplemented use of pre-, pro- and synbiotics for SAP patients due to the presence of limitations.

## Background

Acute pancreatitis (AP) is the most common gastrointestinal condition which is characterized by potentially life-threatening manner (Goldacre and Roberts, 2004). The estimated annual incidence of AP ranges from 13 to 45 cases per 100,000 population worldwide (Forsmark et al., 2016), and in the United States (Peery et al., 2012) and Europe (Britainireland, 2005), the incidence has been increasing by 5% every year. AP has been causing extreme economic burden. For example, it was also responsible for USD $2.6 billion in health-care costs in 2009 in the United States (Peery et al., 2012). As the most serious type of AP, severe acute pancreatitis (SAP) accounts for 15–20% of all AP cases (Banks and Freeman, 2006).

It is believed that necrotic tissue infection is one of the principal causes of complications and death (Deitch, 1990; Ammori et al., 1999; Dervenis et al., 2003), and published studies demonstrated a mortality of 15–25% and a morbidity rate of 50–100% when necrotic tissue was becoming infected (Rodriguez et al., 2008; van Santvoort et al., 2010). To date, failure of the gut barrier and subsequent bacterial translocation and necrotic tissues were regarded as the critical contributor to the infection of necrotic pancreatic tissues (Dervenis et al., 2003; Van Felius et al., 2003; van Minnen et al., 2006). And thus, it may be a potential approach to maintain gut integrity to prevent bacterial and endotoxin translocation and eventually reduce the rate of secondary infection of pancreatic necrosis and decrease mortality and morbidity (Oláh and Romics, 2014).

Published animal experiments (Muftuoglu et al., 2006; van Minnen et al., 2007; Karen et al., 2010) found that probiotics have the potential of maintaining gut integrity and thus minimize bacterial translocation and prevent infection in AP, and a meta-analysis of probiotic supplementation on experimental acute pancreatitis also shown evidence for efficacy (Hooijmans et al., 2012). Moreover, some clinical trials also investigated the potential of probiotics supplementation in critical illness (Sanaie et al., 2014; Rongrungruang et al., 2015; Zeng et al., 2016), and Manzanares et al. (2016) performed a meta-analysis to confirm the efficacy of probiotics supplementation in reducing infection in patients with critical illness. Based on these promising results, some clinical trials (Karakan et al., 2007; Oláh et al., 2007; Besselink et al., 2008; Cui et al., 2013) have been developed to explore the efficacy of supplemented use of pre-, pro-, and synbiotics in patients with SAP and showed beneficial results. However, PROPATRIA trial generated inconsistent findings; that is to say, they found that probiotics supplementation had harmful effects for SAP (Besselink et al., 2008). In order to address the contradictory, two meta-analyses (Sun et al., 2009; Gou et al., 2014) have been performed, and all showed neither beneficial nor adverse effects on the clinical outcomes of patients with predicted SAP. It is surprising that, however, PROPATRIA group carried out an animal experiment to investigate the association between probiotics supplementation and enteral nutrition in an experimental AP model in 2014 and found no negative association between prophylactic probiotics and enteral nutrition in AP (van Baal et al., 2014). Additionally, it is important to emphasize that all previous meta-analyses have some limitations, for example, the trial reported by Oláh et al. (2002), which recruited patients with mild, moderate and severe degrees of pancreatitis, was pooled. Moreover, all meta-analyses did not enroll all potentially eligible trials, and three potential studies (Li et al., 2014; Zhu et al., 2014; Wu et al., 2017) with inconsistent results have been recently published. And thus, we performed the present updated meta-analysis to further investigate the efficacy and safety of supplemented use of pre-, pro- and synbiotics for the treatment of SAP.

## Methods

We designed the present systematic review and meta-analysis in accordance with the criteria recommended by Cochrane Collaboration (Higgins and Green, 2011). We used the preferred items for systematic review and meta-analysis (PRISMA) criteria to guide reporting the results (Moher et al., 2009). We did not need to obtain the informed consent from participants because all analyses in the present study were performed based on published data.

### Selection criteria

We designed the inclusion and exclusion criteria according to the criteria developed by Gou et al. (2014). That is to say, all human RCTs investigated the potential of pre-, pro-, and synbiotics supplementation in SAP patients, who should be definitively diagnosed based on accepted criteria, for example Acute Physiology and Chronic Health Evaluation (APACHE II) score of 8 or more, Imrie/modified Glasgow score of 3 or more, or C-reactive protein over 150 mg/L, will be considered (Gou et al., 2014). Studies will be excluded if the following criteria were met: (1) mild and moderate patients with AP were recruited and SAP patients were not analyzed separately, (2) duplication with poor methodology and insufficient data, and (3) essential data were not reported. We also considered abstract with sufficient data in the present study.

### Outcomes of interesting

We selected infected pancreatic necrosis, mortality, total infection, and length of hospital stay as the primary outcomes, and surgical intervention, systemic inflammatory response syndrome (SIRS), multiple organ failure (MOF), other infectious complications including chest infection, urinary tract infection and septic morbidity, use of antibiotics, and quality of life (QoL) as the secondary outcomes.

### Identification of citations

Two independent reviewers electronically performed a systematic search in PubMed, EMBASE, and Cochrane Central Register of Controlled Trials (CENTRAL) to capture all potential human RCTs investigated the efficacy of pre-, pro-, and synbiotics in patients with SAP from 1992 to January 15, 2018. The following terms including pancreatitis, prebiotics, probiotics, synbiotics, lactobacillus, bifidobacterium, Akkermansia Muciniphila, escherichia, and random were used to construct all search algorithms according to the specific requests of each targeted database. We documented all search algorithms in Supplementary Materials of search algorithm.

We also hand checked the bibliographies of all eligible studies and topic related reviews in order to find any eligible studies. Certainly, we did not impose the language restriction and the publication status so that selection and publication bias can be avoided. In the current stage, any divergences regarding search algorithms and results were solved by consulted a third senior reviewer.

### Data extraction

We assigned two reviewers independently adopted the predesigned the data extraction table (Song et al., 2015) to extract the following information: leading author, publication year, country of leading authors, number sex and age of patients, intervention regimes, and outcomes of interesting. If the essential information were missing, we will contact the corresponding authors of this study to obtain it. If the disagreements about eligibility existed in the two reviewers, a senior third reviewer was consulted in order to get a consensus.

### Quality assessment of eligible individual study

Two independent reviewers used Cochrane risk of bias assessment tool to appraise the risk of bias of each trial (Higgins et al., 2011). According to the recommendations of Cochrane Collaboration (Higgins and Green, 2011), we assessed the six domains including randomization, allocation, blind, incomplete data, selectively reported and other bias. According to the match level between the actual information and the evaluation criteria, a study will be rated as “low risk of bias,” “unclear risk of bias,” and “high risk of bias.” If two reviewers have inconsistent judgment on the risk of bias of each study, a third senior reviewer was invited to address the disagreement. If most of eligible studies were rated to be unclear or low risk of bias, the overall quality was regarded to be moderate.

### Statistical analysis

We performed meta-analysis based on the random effect model, which incorporates within and between studies heterogeneity, to estimate the summarized estimates (DerSimonian and Laird, 1986). For dichotomous data, the pooled estimates were expressed as risk ratio (RR) with 95% confidence intervals (CIs), and the mean difference (MD) with 95% CIs was used to calculate the continuous data (Higgins and Green, 2011). We tested the heterogeneity among all eligible studies for each outcome before performed the meta-analysis. We performed Cochrane Q test (i.e., Chi square method) to qualitatively analyze the heterogeneity (Bowden et al., 2011), and the I2 statistic to quantitatively estimate the proportion of the overall variation that is attributable to between study heterogeneity (Higgins and Thompson, 2002). If I2 statistic >50%, studies were considered as heterogeneous, and in contrast, studies were homogeneous (Higgins and Thompson, 2002). Funnel plot was drawn if the number of studies analyzed in single outcome was more than 10 in order to identify potential publication bias (Palma Perez and Delgado Rodriguez, 2006). If an eligible study has multiple-arm design, we extracted the data from intervention groups which were eligible inclusion criteria according to the recommendations proposed by Cochrane Collaboration (Higgins and Green, 2011). If a study only reported median, range, and sample size, we estimated the mean and variance (standard deviation, SD) based on the method proposed by Hozo et al. (2005). Moreover, we also separately analyzed the value of prebiotics, probiotics or synbiotics alone for primary outcomes in SAP patients by using subgroup analysis method. Certainly, we also test the difference of the role of pre-, pro-, and synbiotics in SAP patients from different regions based on subgroup analysis. We performed all statistical analyses using the RevMan version 5.3 software (Copenhagen, Denmark: The Nordic Cochrane Centre, The Cochrane Collaboration, 2013) (Higgins and Green, 2011).

## Results

### Identification and selection of studies

**Figure 1:**
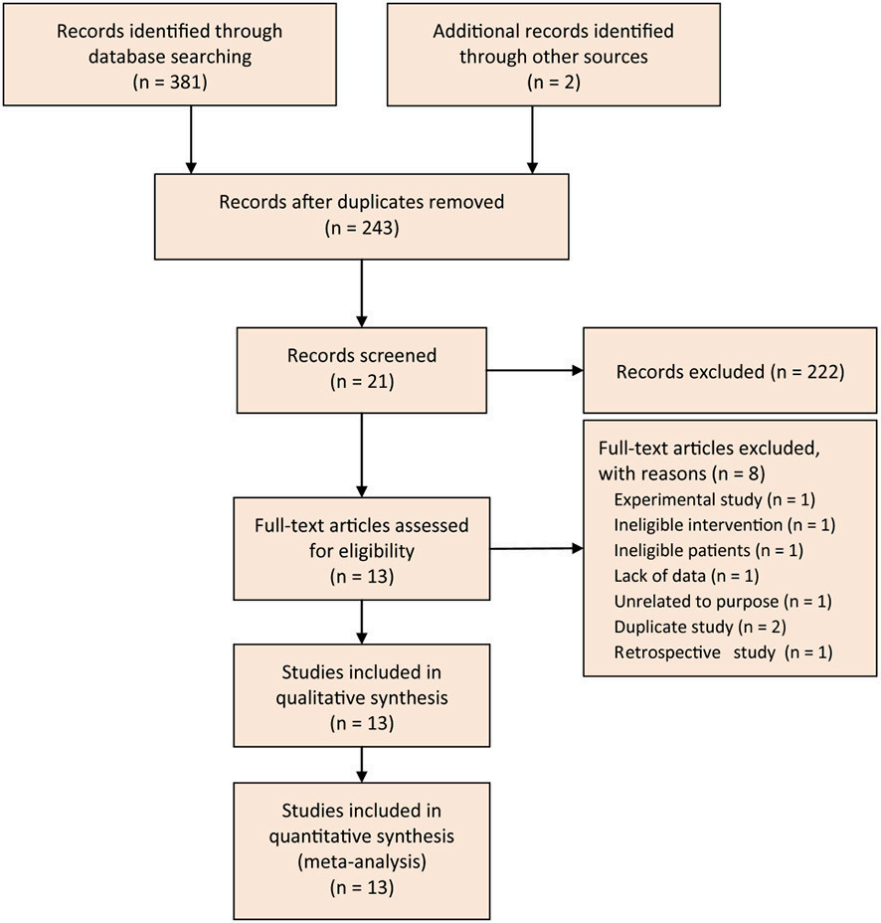
Flow diagram of retrieval and screen of study.

We designed the Figure 1 to depict the process of searching and selecting potential studies. At initial search phase, we captured 381 records in PubMed, EMBASE, and CENTRAL databases. We imported all captured records into the EndNote X7 software, and then constructed the literature database.

We designed a three-step approach to screen and select eligible studies. Firstly, we eliminated 138 records after ran the function of removing duplicates which was embedded in the EndNote software. Secondly, we read the title and abstract of remaining records and excluded 222 citations again: 13 were meta-analysis, 9 were narrative review, 1 was retrospective design, 18 were experimental studies, 1 was correspondence, 4 were the reporting from the same group, 1 was study protocol, 1 was the duplicate, and 138 were unrelated to my topic. Thirdly, we omitted 8 trials after screened the full-text of remaining 21 studies: 1 was experimental study, 1 designed ineligible intervention, 1 recruited ineligible patient, 1 did not report essential data, 1 was unrelated to the given topic, 2 were duplicate studies, and 1 was retrospective design. Eventually, we included 13 eligible trials (Karakan et al., 2007; Li, 2007; Oláh et al., 2007; Besselink et al., 2008; Cui et al., 2009, 2013; Wu and Zhang, 2009; Lata et al., 2010; Plaudis et al., 2012; Wang et al., 2013; Li et al., 2014; Zhu et al., 2014; Wu et al., 2017) to perform the statistical analysis. Of these 13 trials, two (Wu and Zhang, 2009; Li et al., 2014) were added from previous studies.

### Characteristics of eligible studies

We documented the basic characteristics of all 13 eligible studies in Table 1. All 13 studies published between 2007 and 2017, and of which 8 (Li, 2007; Cui et al., 2009, 2013; Wu and Zhang, 2009; Wang et al., 2013; Li et al., 2014; Zhu et al., 2014; Wu et al., 2017) reported in China. A total of 950 patients were recruited and the sample size of a single trial was ranging from 22 to 296 with the median of 49. Of all included studies, three (Plaudis et al., 2012; Wang et al., 2013; Li et al., 2014) were the three-arm design. Only Besselink et al's (2008) trial was multicenter design and remaining 12 were all single center study. Five studies (Li, 2007; Oláh et al., 2007; Cui et al., 2009, 2013; Wu and Zhang, 2009) designed treatment duration of seven days, four studies (Wang et al., 2013; Li et al., 2014; Zhu et al., 2014; Wu et al., 2017) lasted 14 days, and one study (Besselink et al., 2008) was completed after 28 days. Moreover, three studies (Karakan et al., 2007; Lata et al., 2010; Plaudis et al., 2012) did not report the treatment duration clearly. All studies also reported that the baseline of all recruited participants were not significant difference.

**Table 1:** Basic characteristics of all 13 eligible studies.

**Study**	**Country**	**No. of patients (P/C)**	**Sex (P/C, M/F)**	**Age (Probiotics/Control)**	**Characteristics of patients (Probiotics/Control)**	**Intervention (Probiotics/Control)**		**Outcomes**
Karakan et al., 2007	Turkey	15/15	(6/9) vs. (8/7)	47.3 ± 16.8 vs. 44.9 ± 11.2	Mean APACHE II score (9.4/9.6), mean Balthazar CT score (8.5/9.1), mean CRP (232/244) mg/L	0.7 g/100 mL soluble fibers and 0.8 g/100 mL insoluble fibers	Standard enteral nutrition	Infected pancreatic necrosis, organ failure, mortality, total infection, length of hospital stay
Oláh et al., 2007	Hungary	33/29	(27/6) vs. (25/4)	47.5 (19-78) vs. 46.0 (20-81)	Mean Imrie score (2.9/3.1), mean CRP (216.7/191.2) mg/L	10^10^ P. pentosaceus, 10^10^ L. mesenteroides, 10^10^ L. paracasei and 10^10^ L. plantarum with bioactive fibers (Synbiotic 2000 Forte; Medifarm, Kågeröd, Sweden), once daily for 7 days	Bioactive fibers	Infected pancreatic necrosis, organ failure, SIRS, total infection, chest infection, urinary tract infection, mortality, operation, length of hospital stay
Li, 2007	China	14/11	15/10	45 ± 13	APACHE II score 8–20	10^7^ B. longum, 10^6^ L. bulgaricus, and 10^6^ S. thermophilus (Golden Bifid), thrice daily for 7 days	Water	Length of hospital
Besselink et al., 2008	Netherlands	152/144	(91/61) vs. (83/61)	60·4 ± 16·5 vs. 59·0 ± 15·5	Mean APACHE II score (8.6/8.4), mean Imrie score (3.3/3.4), mean CRP (268/270) mg/L	10^10^ L. acidophilus, L. casei, L. salivarius, L. lactis, B. bifidum and B. lactis in a totally daily dose of (Ecologic 641; Winclove Bio Industries, Amsterdam, the Netherlands), twice daily for 28 days	Placebo	Infected pancreatic necrosis, organ failure, SIRS, total infection, chest infection, urinary tract infection, mortality, operation, length of hospital stay
Wu and Zhang, 2009	China	14/13	n.r.	n.r.	APACHE II score 8–20	6 × 10^4^ L. lactis + L. acidophilus and S. lactis	n.r.	Mortality, length of hospital stay
Cui et al., 2009	China	20/25	33/12	45.3 (27–69)	APACHE II score 8–20	10^7^ B. bifidus, B. acidophilus and E. faecalisin (Bifico; Xinyi pharmaceutical factory, Shanghai Pharmaceutical Co., Ltd., China)	Standard enteral nutrition	Infected pancreatic necrosis, organ failure, mortality, length of hospital stay
Lata et al., 2010	Czech Republic	7/15	(3/4) vs. (10/5)	52 ± 12 vs. 55 ± 13	n.a.	B. bifidum, B. infantis, L. acidophilus, L. casei, L. salivarius, L. lactis, twice daily	Placebo	Infected necrosis, Mortality, CRP, Length of hospital stay
Plaudis et al., 2012	Latvia	30/28	n.r.	n.r.	Mean APACHE II score (8.8/8.6)	10^10^ P. pentosaceus, 10^10^ L. mesenteroides, 10^10^ L. paracasei and 10^10^ L. plantarum with bioactive fibers (Synbiotic 2000 Forte), twice daily	Bioactive fibers	Infected pancreatic necrosis, organ failure, SIRS, CRP, total infection, mortality, operation, length of hospital stay
Cui et al., 2013	China	23/25	n.r.	n.r.	APACHE II score 8-20, mean CRP (367.3/384.8) mg/L	4 × 10^7^ B. longum, 4 × 10^7^ L. bulgaricus and 4 × 10^7^ Enterococcus faecalis were supplied twice daily for 14 days	Water	Infected pancreatic necrosis, organ failure, mortality, length of hospital stay
Wang et al., 2013	China	62/61	(32/30) vs. (32/29)	42.6 ± 13.8 vs. 43.7 ± 13.7	Mean APACHE II score (12.89/13.3), mean Ranson score (5.1/5.0), mean CTSI score (6.5/6.7), mean Marshall score (4.9/4.9)	1.5 × 10^7^ B. subtilis and 1.35 × 10^8^ E. faecium entericcoated capsules were supplied thrice daily (Beijing Han Mei Pharmaceutical Company Limited, Beijing, China)	Standard enteral nutrition	Infected pancreatic necrosis, organ failure, mortality
Zhu et al., 2014	China	20/19	(9/11) vs. (9/10)	43.5 ± 17.5 vs. 42.0 ± 16.5	n.r.	2 × 10^7^ C. butyricum (Miyarisan Pharmaceutical Co., Ltd., Nagano, Japan), twice daily for 14 days	Placebo	Infected pancreatic necrosis, urinary tract infection, length of hospital stay
Li et al., 2014	China	27/28	(17/10) vs. (17/11)	49.3 ± 11.5 vs. 47.5 ± 9.6	Mean APACHE II score (11.0/11.1), mean Ranson score (5.1/4.9), mean CRP (384.2/ 378.2) mg/L	Bifidobacterium triple viable including 10^7^ B. bifidus, B, acidophilus and E. faecalis, twice daily	Standard enteral nutrition	Infected pancreatic necrosis, CRP, mortality, length of hospital stay
Wu et al., 2017	China	60/60	(34/26) vs. (32/28)	42.7 ± 11.5 vs. 42.6 ± 13.6	Mean APACHE II score (11.0/11.1), mean Ranson score (5.1/4.9), mean CTSI score (6.6/6.7), mean Marshall score (4.9/4.7)	Bifidobacterium quadruple living bacterium including 0.5 × 10^6^ B. bifidus, 0.5 × 10^6^ B. acidophilus, 0.5 × 10^6^ E. faecalis, and 0.5 × 10^5^ B. cereus, thrice daily	Standard enteral nutrition	Organ failure, total infection, mortality, length of hospital stay

All trials adopted validated criteria to identify the sSAP: APACHE II score ≥ 8 was used in eight studies (Karakan et al., 2007; Li, 2007; Besselink et al., 2008; Cui et al., 2009, 2013; Wu and Zhang, 2009; Li et al., 2014; Wu et al., 2017), Imrie score ≥ 3 was applied in two studies (Oláh et al., 2007; Besselink et al., 2008), Ranson criteria of 3 was adopted in four studies (Cui et al., 2009, 2013; Li et al., 2014; Wu et al., 2017), C-reactive protein (CRP) level in excess of 150 mg/L was used in four studies (Karakan et al., 2007; Oláh et al., 2007; Besselink et al., 2008; Lata et al., 2010), computed tomography (necrosis > 30%) was in five studies (Oláh et al., 2007; Cui et al., 2009, 2013; Li et al., 2014; Wu et al., 2017), and Marshall sub scores ≥ 2 was used in one study (Zhu et al., 2014). Moreover, two studies (Plaudis et al., 2012; Wang et al., 2013) applied the APACHE II score ≥ 6 with SIRS and/or organ dysfunction to define SAP.

In all included studies, 22 strains of probiotic bacteria were used. Moreover, one study (Karakan et al., 2007) used prebiotic, and two studies (Oláh et al., 2007; Plaudis et al., 2012) used the synbiotics. One study (Zhu et al., 2014) only used single strain of probiotic bacteria. One study (Wang et al., 2013) used two strains of probiotic bacteria. Five studies (Li, 2007; Cui et al., 2009, 2013; Wu and Zhang, 2009; Li et al., 2014) used three strains of probiotic bacteria. Three studies (Oláh et al., 2007; Plaudis et al., 2012; Wu et al., 2017) used four strains of probiotic bacteria. Two studies (Besselink et al., 2008; Lata et al., 2010) used six strains of probiotic bacteria.

### Risk of bias of eligible studies

**Figure 2:**
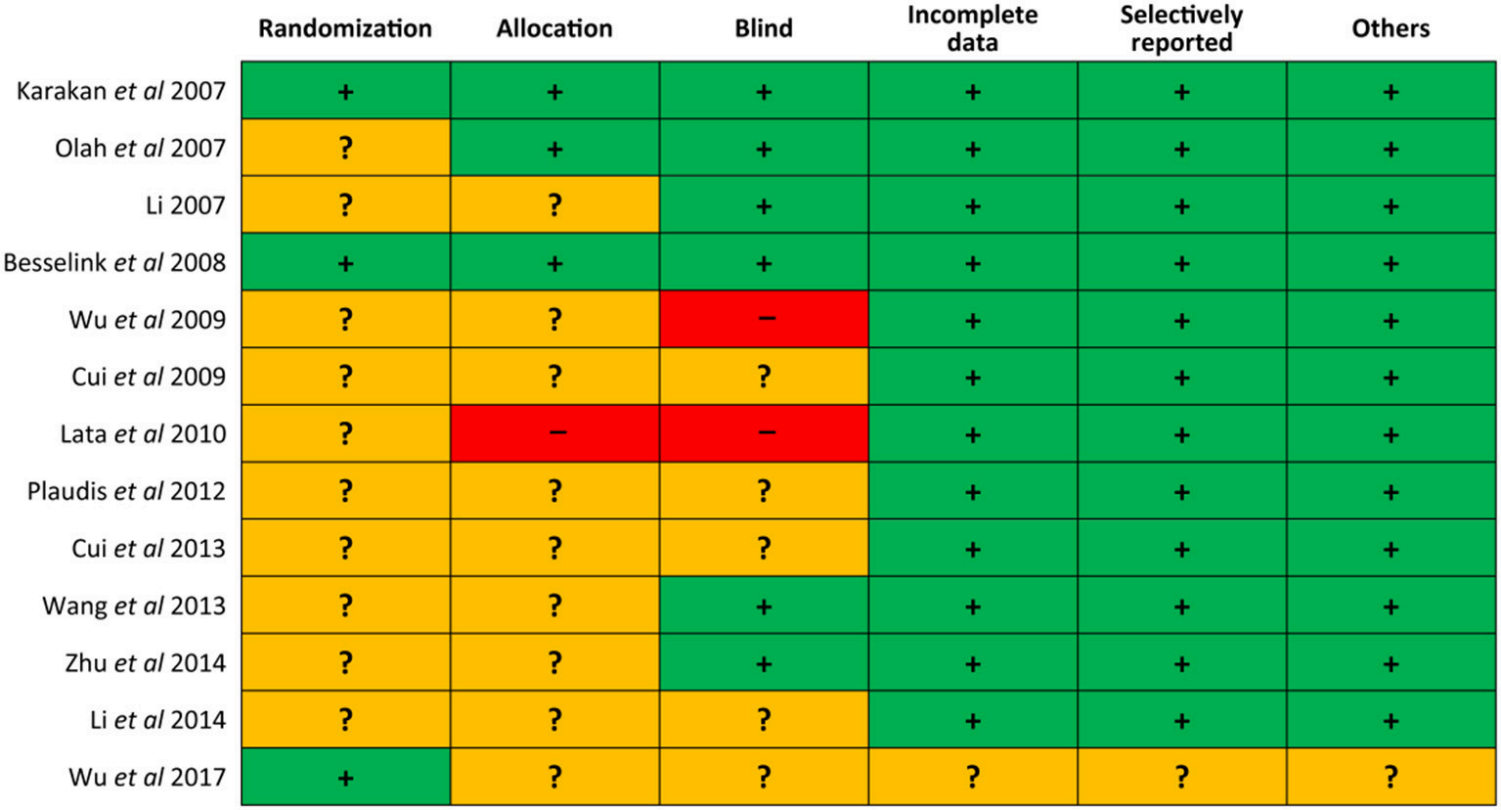
Risk of bias of all 13 eligible studies.

We draw the Figure 2 to delineate the risk of bias of each eligible study. Of 13 trials, three (Karakan et al., 2007; Besselink et al., 2008; Wu et al., 2017) utilized the computerized process to generate random sequence, three (Karakan et al., 2007; Oláh et al., 2007; Besselink et al., 2008) appropriately allocated the patients into each group by using numbered containers, numbered sachet and permuted-block sequence respectively, one (Lata et al., 2010) exposed the concealment because six participants were allocated directly to placebo group for safety reason, six (Karakan et al., 2007; Li, 2007; Oláh et al., 2007; Besselink et al., 2008; Wang et al., 2013; Zhu et al., 2014) correctly carried out blind and two (Wu and Zhang, 2009; Lata et al., 2010) did not perform blind, only one (Wu et al., 2017) was judged has unclear risk of bias in terms of incomplete data, selectively reporting, and other bias. In general, the overall quality of included trials was moderate.

### Primary outcomes

#### Infected pancreatic necrosis

**Figure 3:**
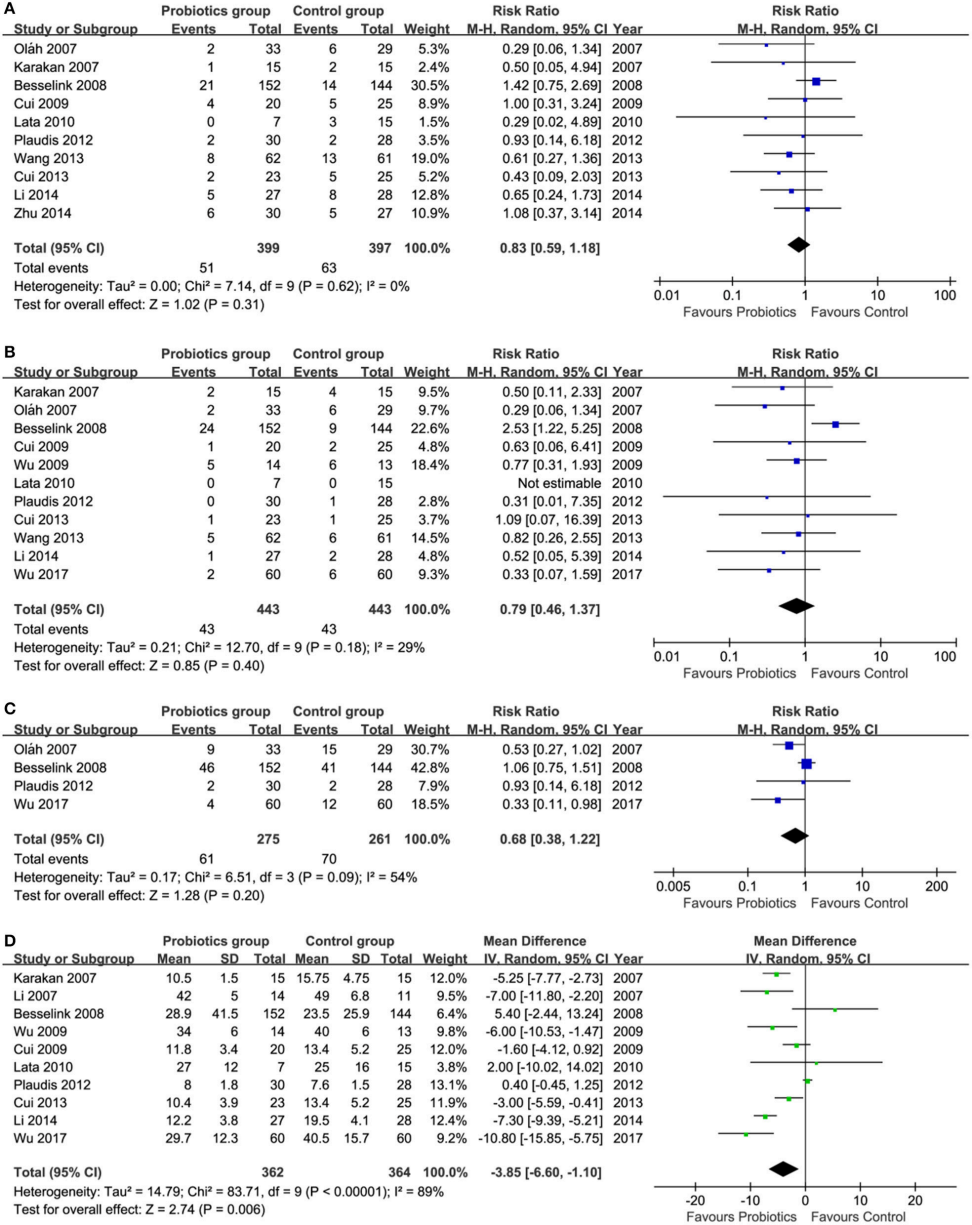
Meta-analysis on primary outcomes. **(A)** infected pancreatic necrosis, **(B)** mortality, **(C)** total infection, and **(D)** length of hospital stay. CI, Confidence interval; M-H, Mantel-Haenszel.

Of these 13 included trials, 10 (Karakan et al., 2007; Oláh et al., 2007; Besselink et al., 2008; Cui et al., 2009, 2013; Lata et al., 2010; Plaudis et al., 2012; Wang et al., 2013; Li et al., 2014; Zhu et al., 2014) reported the infected pancreatic necrosis. Meta-analysis showed a promising trend which was benefited to pre-, pro- and synbiotics group although the difference in reducing infected pancreatic necrosis was not statistically significant (*RR* = 0.83, 95% *CI* = 0.59–1.18; *P* = 0.31; *I*^2^ = 0%) between pre-, pro-, and synbiotics and control groups (Figure 3A).

#### Mortality

Eleven (Karakan et al., 2007; Oláh et al., 2007; Besselink et al., 2008; Cui et al., 2009, 2013; Wu and Zhang, 2009; Lata et al., 2010; Plaudis et al., 2012; Wang et al., 2013; Li et al., 2014; Manzanares et al., 2016; Wu et al., 2017) of all eligible studies reported the mortality. Pooled result suggested that the mortality rate in pre-, pro-, and synbiotics group had the promising trend compared to control group, however, the difference between these two groups did not get statistically significant (*RR* = 0.79, 95% *CI* = 0.46–1.37; *P* = 0.40; *I*^2^ = 29%; Figure 3B).

#### Total infection

The data of total infection can be extracted from four eligible studies (Oláh et al., 2007; Besselink et al., 2008; Plaudis et al., 2012; Wu et al., 2017). Summarized results shown that the difference in controlling total infection events between groups was not significant, but a promising trend which was benefited to pre-, pro- and synbiotics was generated (*RR* = 0.68, 95% *CI* = 0.38–1.22; *P* = 0.20; *I*^2^ = 54%;Figure 3C).

#### Length of hospital stay

Ten eligible studies (Karakan et al., 2007; Li, 2007; Besselink et al., 2008; Cui et al., 2009, 2013; Wu and Zhang, 2009; Lata et al., 2010; Plaudis et al., 2012; Li et al., 2014; Wu et al., 2017) investigated the comparative efficacy of pre-, pro-, and synbiotics related to control group in terms of length of hospital stay. Meta-analysis showed a reduction in length of hospitalization in pre-, pro-, and synbiotics group, with statistically significant difference (*MD* = −3.85, 95% *CI* = −6.60 to −1.10; *P* = 0.006; *I*^2^ = 89%; Figure 3D).

#### Secondary outcomes

Of all 13 eligible studies, three (Oláh et al., 2007; Besselink et al., 2008; Plaudis et al., 2012) reported the surgical intervention and meta-analysis did not showed a statistical difference between two groups (*RR* = 1.18, 95% *CI* = 0.48–2.86; *P* = 0.72; *I*^2^ = 52%; Figure S1A). Meta-analysis based on two studies shown that the different between pre-, pro-, and synbiotics and control groups in terms of SIRS was no significance (*RR* = 0.77, 95% *CI* = 0.16–3.70; *P* = 0.74; *I*^2^ = 82%; Figure S1B). Eight studies reported organ failure and meta-analysis showed no statistically significant difference between pre-, pro- and synbiotics and control groups (*RR* = 0.71, 95% *CI* = 0.46–1.09; *P* = 0.12; *I*^2^ = 69%; Figure S1C). Four studies reported the total number of SIRS and organ failure, and pooled analysis did not generate statistically significant finding (*RR* = 0.96, 95% *CI* = 0.42–2.21; *P* = 0.93; *I*^2^ = 87%; Figure S1D). Six studies reported other infectious complications including chest infection (2 RCTs), urinary tract infection (3 RCTs) and septic morbidity (3 RCTs). Pooled results showed no significant difference between pre-, pro- and synbiotics and control groups in terms of these three given outcomes (Figure S1E). Only one study reported use of antibiotics, and the result suggested no significant difference (Figure S1F). No eligible study reported quality of life (QoL), and thus we did not obtain the summarized finding.

#### Subgroup analysis for primary outcomes

We performed subgroup analysis to investigate the value of prebiotics, probiotics or synbiotics alone with regard to primary outcomes in SAP patients. These all pooled results were consistent with the previous results, which were generated based on all interventions. That is to say, pre- and probiotics were still associated with shorten of length of hospitalization. However, the value of synbiotics in reducing length of hospital stay remains debate due to insufficient number of eligible trials (only one) (Plaudis et al., 2012; Figure S2).

We also performed subgroup analysis to investigate the value of pre-, pro-, and synbiotics in SAP patients from different regions, which were divided into China and western countries. Subgroup analyses generated consistent results with previous analyses with regard to infected pancreatic necrosis and mortality. It is noted that supplemented use of pre-, pro-, and synbiotics showed promising results in terms of total infection (1 RCT, *RR* = 0.33, 95% *CI* = 0.11–0.98, *P* = 0.04) and length of hospital stay (6 RCTs, *MD* = −5.57, 95% *CI* = −8.21 to −2.93, *P* < 0.001) in Chinese patients, however no significant results were not detected in western patients with regard to total infection (3 RCTs, *RR* = 0.82, 95% *CI* = 0.49–1.39, *P* = 0.47) and length of hospital stay (4 RCTs, *MD* = −0.5, 95% *CI* = −4.93 to 3.91, *P* = 0.82).

#### Publication bias

The number of eligible studies of three outcomes including infected pancreatic necrosis, mortality and length of hospital stay met the criteria of performing publication bias. We obtained asymmetric funnel plots in terms of these three outcomes, which indicated a risk of presentence of publication bias (Figure S3).

## Discussion

In the present updated systematic review and meta-analysis of 13 RCTs that compared pre-, pro-, or synbiotics with control regimes in patients with SAP, we found that pre-, pro-, or synbiotics, in a manner, shorten the length of hospital stay (*MD* = −3.85, 95% *CI* = −6.60 to −1.10), however, evidence which was supported to be harmful or beneficial to pre-, pro-, or synbiotics with regard to remaining important clinical outcomes was not detected. It must be noted that, however, we just found promising results in Chinese SAP patients with regard to total infection (*RR* = 0.33, 95% *CI* = 0.11–0.98, *P* = 0.04) and length of hospital stay (*MD* = −5.57, 95% *CI* = −8.21 to −2.93, *P* < 0.001).

Gut barrier integrity, which can prevent bacteria translocation and reduce systemic inflammatory syndrome, plays a critical role in development and progress of SAP (Deitch, 1990), and thus, the major goal of treating SAP is to maintain gut barrier integrity (Sun et al., 2009). Because of no beneficial efficacy of antibiotics prophylaxis for SAP (Mazaki et al., 2006; Vries et al., 2007), researchers and practitioners changed to pay more attentions on potential of pre-, pro-, or synbiotics (Oláh et al., 2002, 2007; Karakan et al., 2007; Li, 2007; Besselink et al., 2008; Cui et al., 2009, 2013; Wu and Zhang, 2009; Lata et al., 2010; Plaudis et al., 2012; Wang et al., 2013; Li et al., 2014; Zhu et al., 2014; Wu et al., 2017). Based on these published clinical trials that investigated the efficacy and safety of pre-, pro-, synbiotics for SAP, several meta-analyses (Petrov et al., 2009; Sun et al., 2009; Zhang et al., 2010; Gou et al., 2014; Poropat et al., 2015; Moggia et al., 2017) have also been performed (Table 2).

**Table 2:** Meta-analyses of pre-, pro-, and synbiotics in severe acute pancreatitis.

**Items**	**Sun et al., 2009**	**Petrov et al., 2009**	**Zhang et al., 2010**	**Gou et al., 2014**	**Poropat et al., 2015**	**Moggia et al., 2017**	**The present study**
No. of RCTs	4	3	5	6	6	2	13
No. of participants	428	390	440	536	666	101	950
Search strategy until (year)	2008	2009	2010	2013	2013	2016	2018
**OUTCOMES**							
Infected pancreatic necrosis	RR, 0.56 (0.13, 2.35)	n.r.	RR, 1.06 (0.58, 1.96)	RR, 1.25 (0.79, 1.98)	RR, 0.69 (0.46, 1.05)	RR, 0.60 (0.22, 1.68)	RR, 0.83 (0.59, 1.18)
Mortality	RR, 0.83 (0.14, 4.83)	RR, 0·96 (0·12, 7·83)	RR, 0.77 (0.22, 2.72)	RR, 0.72 (0.42, 1.45)	RR, 1.13 (0.66, 1.91)	RR, 0.25 (0.05, 1.34)	RR, 0.79 (0.46, 1.37)
Total infection	n.r.	RR, 0·79 (0·40, 1·56)	n.r.	RR, 1.09 (0.80, 1.48)	n.r.	n.r.	RR, 0.68 (0.38, 1.22)
Surgical intervention	RR, 0.59 (0.11, 3.07)	n.r.	RR, 1.07 (0.23, 4.92)	RR, 1.42 (0.43, 3.47)	n.r.	n.r.	RR, 1.18 (0.48, 2.86)
Length of hospital stay	MD, 1.20 (−13.33, 10.92)	n.r.	n.r.	MD, 2.45 (−2.71, 7.60)	MD,−1.71 (−6.04, 2.61)	n.r.	***MD, 3.85 (**−**6.60**,−**1.10)***
SIRS	n.r.	n.r.	n.r.	n.r.	RR, 1.07 (0.90, 1.27)	n.r.	RR, 0.77 (0.16, 3.70)
MOF	n.r.	n.r.	n.r.	n.r.	RR, 0.84 (0.67, 1.04)	RR, 0.40 (0.12, 1.36)	RR, 0.71 (0.46, 1.09)
MOF and SIRS	n.r.	n.r.	RR, 0.65 (0.09, 4.44)	n.r.	n.r.	n.r.	RR, 0.96 (0.42, 2.21)
Chest infection	n.r.	n.r.	n.r.	n.r.	n.r.	n.r.	RR, 1.02 (0.36, 2.88)
Urinary tract infections	n.r.	n.r.	n.r.	n.r.	n.r.	n.r.	RR, 0.92 (0.30, 2.82)

*Bold and italic value represents statistical significance*.

Sun et al. (2009) performed the first meta-analysis to investigate the potential of probiotics in patients with SAP, and found that enteral feeding supplemented with probiotic could not reduce the infected necrosis and mortality. It is noted that, however, only 4 RCTs involving 428 patients were analyzed. More importantly, these authors included a RCTs that recruited mild, moderate and severe degrees of pancreatitis patients (Oláh et al., 2002), but did not a RCT performed in 2007 (Karakan et al., 2007). In 2009, Petrov et al. performed a systematic review and meta-analysis with regard to enteral nutrition formulations in AP (Petrov et al., 2009), in which these authors designed a subgroup analysis targeted to SAP, and no significant differences in feeding intolerance, total infectious complication, and mortality were detected when fiber enriched plus probiotics versus fiber enriched. Similarly, this meta-analysis only enrolled three RCTs with 390 patients, and also considered the RCT reported by Oláh et al. (2002) but did not two RCT carried in 2009 (Cui et al., 2009; Wu and Zhang, 2009). Zhang et al. designed meta-analysis to determine the value of pre-, pro-, and synbiotics in AP in 2010 (Zhang et al., 2010). In which, these authors also analyzed the data of SAP using subgroup method, and found that pre-, pro-, and synbiotics had no significant influence on the main surgical outcomes. The power of findings from this meta-analysis was higher than previous meta-analyses due to more sample size and reasonably eligible RCTs. However, a limitation that two RCTs performed by Cui et al. (2009) and Lata et al. (2010) respectively in 2010 were not enrolled impaired the power of this meta-analysis. Guo et al. performed a well-designed meta-analysis of investigated use of probiotics in the treatment of SAP (Gou et al., 2014). In this meta-analysis, authors included six eligible RCTs with 536 patients, and concluded that probiotics showed neither beneficial nor adverse effects on the clinical outcomes. Unfortunately, the study also considered the RCT reported by Oláh et al. (2002), and moreover, four potential RCTs (Cui et al., 2009; Wu and Zhang, 2009; Lata et al., 2010; Wang et al., 2013) were not incorporated. And thus, these pooled results from this meta-analysis should be cautiously interpreted. In 2015 and 2017, Poropat et al. (2015) and Moggia et al. (2017) performed one Cochrane review to assess the beneficial and harmful effects of different enteral nutrition formulations and different pharmacological interventions in patients with AP respectively. In these two reviews, subgroup analysis of SAP was also designed. According to pooled results, significant difference with regard to clinical outcomes was also not generated. However, Poropat et al.'s review considered that trial performed by Oláh et al. (2002), and five potentially eligible RCTs were not enrolled (Li, 2007; Cui et al., 2009, 2013; Wu and Zhang, 2009; Moggia et al., 2017). For Moggia et al.'s review, only two eligible studies were included. Compared to previous meta-analyses, the present study included 13 eligible RCTs with 950 participants. Moreover, we analyzed 13 important clinical outcomes which were not studied completely in previous studies, and found that pre-, pro-, and synbiotics reduced length of hospital stay in patients with SAP. More importantly, subgroup analysis in our study also suggested an association between probiotics and reduction of length of hospital stay. At the same time, we also found a promising trend which was beneficial to pre-, pro-, and synbiotics group in terms of several outcomes such as infected pancreatic necrosis and mortality.

Although a RCT using single strain of *C. butyricum* in 2014 revealed that probiotics did not reduce infectious complications, shorten the length of intensive care stay, but increased the rate of intestinal ischemia and necrosis (Zhu et al., 2014), two recent RCTs (Li et al., 2014; Wu et al., 2017) using multiple strains of probiotic bacteria supported that probiotics reduced the incidence of infection, organ failure and mortality and shorten the length of hospital stay. It is noted that published studies (Meijerink et al., 2010; Macho et al., 2011) suggested that the different strain of probiotic bacteria may exert different performance. And thus, we had to suppose that mixture of validated strains of probiotic bacteria will be beneficial to SAP. Interestingly, although PROPATRIA group showed that probiotics had harmful effects in 2008, they did also established no negative association between prophylactic probiotics and enteral nutrition in AP based on an experimental AP rats model in 2014 (van Baal et al., 2014). It must be noted that two trials that investigated the potential of probiotic mixtures which were similar to probiotic regime of PROPATRIA trial in critical illness showed that probiotics had a detrimental effect on infection (Jain et al., 2004; Barraud et al., 2010), however, partial of these six strains in other two trials (Wu and Zhang, 2009; Lata et al., 2010) did generated beneficial results. Hence, the performance of mixture of different strains of probiotic bacteria should be further explored. Moreover, PROPATRIA trial designed treatment duration of 28 days, and Guo et al. tentatively supposed that prolonged treatment duration may lead to an overload of probiotics, which might be harmful to patients with SAP and critical illness who have intestinal barrier dysfunction (Gou et al., 2014). And thus, we also considered that the findings from PROPATRIA trial may be questionable due to prolonged treatment duration. More importantly, a retrospective study (Van Baal et al., 2011) revealed that probiotic treatment had no apparent negative effect on patients with predicted SAP patients without initial organ failure, and another involved 79 patients with severe acute pancreatitis did found a reduction in length of hospitalization, infectious complications and organ failure (Liu et al., 2015).

The present meta-analysis generated more reliable findings based on relatively large eligible studies and cumulated sample size and rigorous method of addressing data; however some limitations must be interpreted. Firstly, the present study simultaneously considered potential of pre-, pro-, and synbiotics in patients with SAP, however previous studies just included probiotics. Nevertheless, we also investigated the effect of pre-, pro- and synbiotics alone on primary outcomes, and generated similar results. Secondly, we did not perform subgroup analysis according to the strain of probiotic bacteria because information of single strain was not available in most of included trials. And thus, further studies should consider the effect and safety of different strains of probiotic bacteria. Thirdly, we did not design subgroup to investigate the impact of treatment duration on effects and safety because three trials did not clearly report the time of intervention. Although it is unclear that whether the treatment duration can affect the potential of pre-, pro-, and synbiotics, however subgroup analysis designed in Guo et al.'s meta-analysis suggested that prolonged duration of treatment might be harmful to patients with SAP and critical illness who have intestinal barrier dysfunction. And thus, we suggested more well-designed trials to confirm the association between duration of treatment and efficacy and safety of pre-, pro- and synbiotics. Fourthly, the present did not prospectively register in internal platform. Fifthly, we obtained different results with regard to total infection when subgroup analysis was performed based on region. That is to say, Chinese SAP patients benefited from supplemented use of pre-, pro-, and synbiotics, however western patients did not. In fact, a point must be emphasized, for total infection, only one eligible RCT was analyzed and thus we suppose that the result may be affected largely by the small sample size. Finally, the shortening of the length of hospital stay through the administration of pre-, pro- and synbiotics is limited to publications with Chinese cohorts (sub-analyses). Certainly, although the pooled results from Chinese and Western SAP patients were inconsistent, the result based on all patients generated promising finding, and thus we speculate that the inadequate sample size was the important contributor to the difference. Consequently, it is essential to develop further large study investigating the role of pre-, pro-, and synbiotics supplementation in SAP patients from different regions.

## Conclusions

Although the accumulated sample size of 950 was still not sufficient, the present pooled results still indicated that pre-, pro-, or synbiotics, in a manner, shorten the length of hospital stay in Chinese SAP patients. Moreover, the findings from our study also indicated a promising trend which was benefited to pre-, pro-, or synbiotics with regard to most of other clinical outcomes such as infected pancreatic necrosis and mortality. And thus, we concluded that pre-, pro-, or synbiotics may have potential for the treatment of SAP. Nevertheless, it is essential to design RCTs with large-scale and appropriate blinded method to further establish the efficacy and safety of pre-, pro-, or synbiotics in SAP before making recommendations due to the presence of limitations. Moreover, more experimental studies should also be designed in order to deeply explore the mechanisms of pre-, pro-, or synbiotics in SAP.

## Clinical relevancy statement

The potential of pre-, pro-, and synbiotics supplemented to standard enteral nutrition in patients with severe acute pancreatitis is uncertain. Our updated systematic review and meta-analysis based on 13 RCTs describes the benefit that pre-, pro-, and synbiotics, in a manner, shortened the length of hospital stay in Chinese SPA patients. And thus, our findings are both essential and clinically relevant to provide patients with severe acute pancreatitis to receive enteral nutrition supplemented with pre-, pro-, and synbiotics.

## Author contributions

XT and W-QC conceived and designed the study. XT, Y-PP, X-LL, and HC participated in study selection, data extraction. XT, Y-PP, and X-LL performed statistical analysis. XT and HC were involved in manuscript drafting and revision. All authors approved the final manuscript for submission and publication.

### Conflict of interest statement

The authors declare that the research was conducted in the absence of any commercial or financial relationships that could be construed as a potential conflict of interest.
