# Supplementary material for: Supplemented Use of Pre-, Pro-, and Synbiotics in Severe Acute Pancreatitis: An Updated Systematic Review and Meta-Analysis of 13 Randomized Controlled Trials
Source: Front Pharmacol. 2018 Jun 28;9:690. doi: 10.3389/fphar.2018.00690 (PMC6031870; doi:10.3389/fphar.2018.00690)
Supplement: Supplementary file 1 [file Table_1.PDF]

**Recent queries in pubmed**	
Search	Query
#8	Search (((((("Pancreatitis"[Mesh] OR "Pancreatitis, Acute Necrotizing"[Mesh] OR "Pancreatitis, Alcoholic"[Mesh]))) OR Pancreatitis)) AND ((((((("Probiotics"[Mesh]) OR "Prebiotics"[Mesh]) OR "Synbiotics"[Mesh]) OR "Bifidobacterium"[Mesh]) OR "Escherichia"[Mesh])) OR (((((((probiotic*) OR prebiotic*) OR synbiotic*) OR Lactobacill*) OR Bifidobacterium*) OR Akkermansia Muciniphila) OR Escherichia))) AND random*
#7	Search random* Sort by: PublicationDate
#6	Search (((((("Probiotics"[Mesh]) OR "Prebiotics"[Mesh]) OR "Synbiotics"[Mesh]) OR "Bifidobacterium"[Mesh]) OR "Escherichia"[Mesh])) OR (((((((probiotic*) OR prebiotic*) OR synbiotic*) OR Lactobacill*) OR Bifidobacterium*) OR Akkermansia Muciniphila) OR Escherichia) Sort by: PublicationDate
#5	Search ((((((probiotic*) OR prebiotic*) OR synbiotic*) OR Lactobacill*) OR Bifidobacterium*) OR Akkermansia Muciniphila) OR Escherichia Sort by: PublicationDate
#4	Search (((("Probiotics"[Mesh]) OR "Prebiotics"[Mesh]) OR "Synbiotics"[Mesh]) OR "Bifidobacterium"[Mesh]) OR "Escherichia"[Mesh] Sort by: PublicationDate
#3	Search ((("Pancreatitis"[Mesh] OR "Pancreatitis, Acute Necrotizing"[Mesh] OR "Pancreatitis, Alcoholic"[Mesh]))) OR Pancreatitis Sort by: PublicationDate
#2	Search Pancreatitis Sort by: PublicationDate
#1	Search ("Pancreatitis"[Mesh] OR "Pancreatitis, Acute Necrotizing"[Mesh] OR "Pancreatitis, Alcoholic"[Mesh]) Sort by: PublicationDate

Recent queries in EMBASE	
**No.**	**Query**
#9	#4 AND #7 AND #8
#8	random*
#7	#5 AND #6
#6	probiotic* OR 'prebiotic'/exp OR prebiotic OR synbiotic* OR lactobacill* OR bifidobacterium* OR (('akkermansia'/exp OR akkermansia) AND muciniphila) OR 'escherichia'/exp OR escherichia
#5	'probiotic agent'/exp OR 'probiotic agent' OR 'prebiotic agent'/exp OR 'prebiotic agent' OR 'synbiotic agent'/exp OR 'synbiotic agent' OR 'bifidobacterium'/exp OR bifidobacterium OR 'escherichia'/exp OR escherichia OR 'lactobacillus'/exp OR lactobacillus
#4	#1 OR #2 OR #3
#3	'alcoholic pancreatitis'/exp OR 'alcoholic pancreatitis'
#2	'acute hemorrhagic pancreatitis'/exp OR 'acute hemorrhagic pancreatitis'
#1	'pancreatitis'/exp OR pancreatitis

Recent queries in CENTRAL	
**No.**	**Query**
#1	Pancreatitis:ti,ab,kw (Word variations have been searched)
#2	MeSH descriptor: [Pancreatitis] explode all trees
#3	"probiotic":ti,ab,kw or prebioitic*:ti,ab,kw or synbiotic*:ti,ab,kw or Bifidobacterium*:ti,ab,kw or Lactobacill*:ti,ab,kw (Word variations have been searched)
#4	Akkermansia Muciniphila:ti,ab,kw or Escherichia:ti,ab,kw (Word variations have been searched)
#5	MeSH descriptor: [Probiotics] explode all trees
#6	MeSH descriptor: [Prebiotics] explode all trees
#7	MeSH descriptor: [Synbiotics] explode all trees
#8	MeSH descriptor: [Bifidobacterium] explode all trees
#9	MeSH descriptor: [Escherichia] explode all trees
#10	#1 or #2
#11	#3 or #4 or #5 or #6 or #7 or #8 or #9
#12	random*:ti,ab,kw (Word variations have been searched)
#13	#10 and #11 and #12
