# Supplementary material for: Supplemented Use of Pre-, Pro-, and Synbiotics in Severe Acute Pancreatitis: An Updated Systematic Review and Meta-Analysis of 13 Randomized Controlled Trials
Source: Front Pharmacol. 2018 Jun 28;9:690. doi: 10.3389/fphar.2018.00690 (PMC6031870; doi:10.3389/fphar.2018.00690)
Supplement: Supplementary file 2 [file Table_2.PDF]

A

Study or Subgroup	Probiotics group Events	Probiotics group Total	Control group Events	Control group Total	Weight	Risk Ratio M-H, Random, 95% CI	Year
Oláh 2007	4	33	7	29	31.6%	0.50 [0.16, 1.54]	2007
Besselink 2008	28	152	14	144	49.4%	1.89 [1.04, 3.45]	2008
Plaudis 2012	3	30	2	28	19.0%	1.40 [0.25, 7.77]	2012
**Total (95% CI)**		**215**		**201**	**100.0%**	**1.18 [0.48, 2.86]**	
Total events	35		23				

Heterogeneity: Tau² = 0.32; Chi² = 4.18, df = 2 (P = 0.12); I² = 52%
Test for overall effect: Z = 0.36 (P = 0.72)

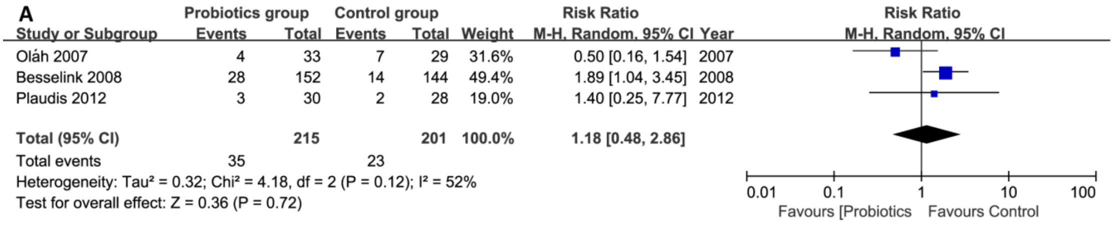

B

Study or Subgroup	Probiotics group Events	Probiotics group Total	Control group Events	Control group Total	Weight	Risk Ratio M-H, Random, 95% CI
Oláh 2007	3	33	5	29	41.2%	0.53 [0.14, 2.02]
Plaudis 2012	30	30	28	28	58.8%	1.00 [0.94, 1.07]
**Total (95% CI)**		**63**		**57**	**100.0%**	**0.77 [0.16, 3.70]**
Total events	33		33			

Heterogeneity: Tau² = 1.09; Chi² = 5.66, df = 1 (P = 0.02); I² = 82%
Test for overall effect: Z = 0.33 (P = 0.74)

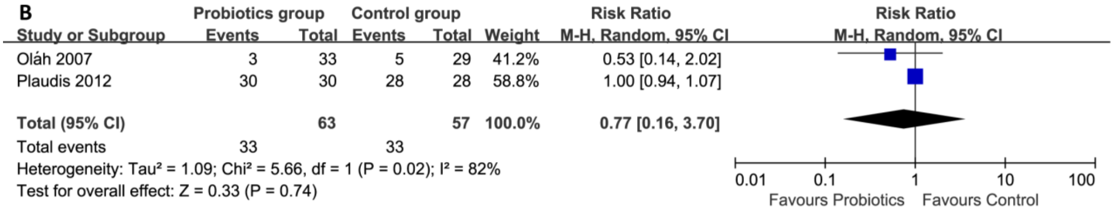

C

Study or Subgroup	Probiotics group Events	Probiotics group Total	Control group Events	Control group Total	Weight	Risk Ratio M-H, Random, 95% CI	Year
Oláh 2007	6	33	14	29	12.2%	0.38 [0.17, 0.85]	2007
Karakan 2007	1	15	2	15	3.1%	0.50 [0.05, 4.94]	2007
Besselink 2008	21	152	16	144	15.1%	1.24 [0.68, 2.29]	2008
Cui 2009	10	20	10	25	14.5%	1.25 [0.65, 2.39]	2009
Plaudis 2012	27	30	24	28	20.7%	1.05 [0.87, 1.27]	2012
Cui 2013	4	23	10	25	9.9%	0.43 [0.16, 1.20]	2013
Wang 2013	7	62	15	61	12.1%	0.46 [0.20, 1.05]	2013
Wu 2017	7	60	16	60	12.3%	0.44 [0.19, 0.99]	2017
**Total (95% CI)**		**395**		**387**	**100.0%**	**0.71 [0.46, 1.09]**	
Total events	83		107				

Heterogeneity: Tau² = 0.23; Chi² = 22.61, df = 7 (P = 0.002); I² = 69%
Test for overall effect: Z = 1.55 (P = 0.12)

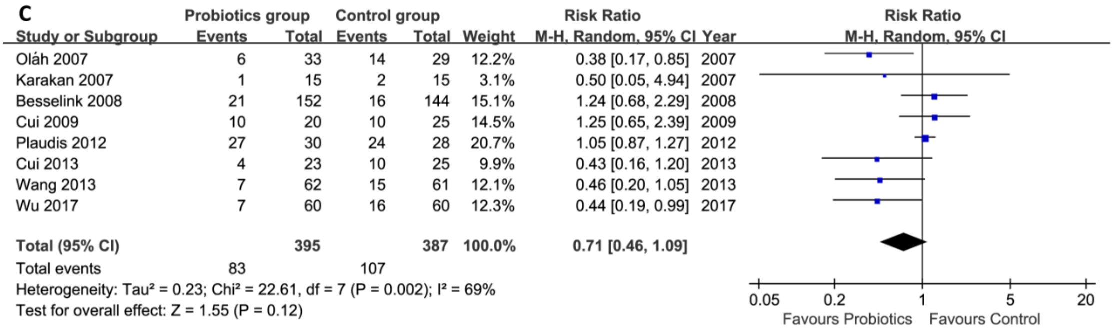

D

Study or Subgroup	Probiotics group Events	Probiotics group Total	Control group Events	Control group Total	Weight	Risk Ratio M-H, Random, 95% CI
Besselink 2008	33	152	15	144	29.4%	2.08 [1.18, 3.67]
Karakan 2007	1	15	2	15	9.4%	0.50 [0.05, 4.94]
Oláh 2007	8	33	14	29	27.2%	0.50 [0.25, 1.02]
Plaudis 2012	30	30	28	28	34.0%	1.00 [0.94, 1.07]
**Total (95% CI)**		**230**		**216**	**100.0%**	**0.96 [0.42, 2.21]**
Total events	72		59			

Heterogeneity: Tau² = 0.52; Chi² = 23.94, df = 3 (P < 0.0001); I² = 87%
Test for overall effect: Z = 0.09 (P = 0.93)

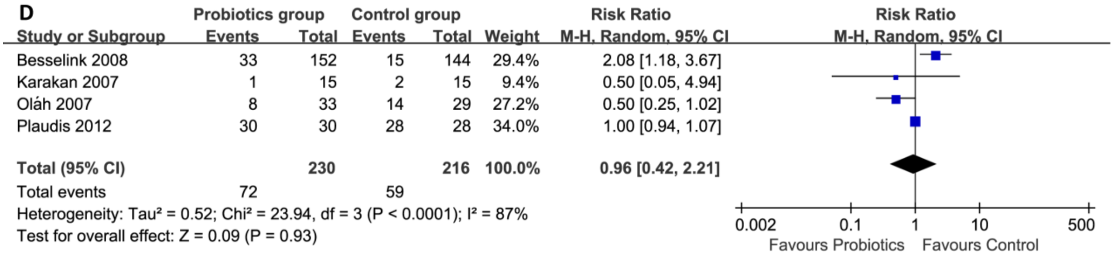

E

Study or Subgroup	Probiotics group Events	Probiotics group Total	Control group Events	Control group Total	Weight	Risk Ratio M-H, Random, 95% CI	Year
**Chest infection**							
Oláh 2007	2	33	4	29	6.3%	0.44 [0.09, 2.23]	2007
Besselink 2008	24	152	16	144	47.4%	1.42 [0.79, 2.56]	2008
**Subtotal (95% CI)**		**185**		**173**	**53.7%**	**1.02 [0.36, 2.88]**	
Total events	26		20				
Heterogeneity: Tau² = 0.30; Chi² = 1.78, df = 1 (P = 0.18); I² = 44%							
Test for overall effect: Z = 0.03 (P = 0.97)							
**Urinary tract infection**							
Oláh 2007	3	33	3	29	7.1%	0.88 [0.19, 4.02]	2007
Besselink 2008	1	152	2	144	2.9%	0.47 [0.04, 5.17]	2008
Zhu 2014	2	20	1	19	3.1%	1.90 [0.19, 19.27]	2014
**Subtotal (95% CI)**		**205**		**192**	**13.1%**	**0.92 [0.30, 2.82]**	
Total events	6		6				
Heterogeneity: Tau² = 0.00; Chi² = 0.68, df = 2 (P = 0.71); I² = 0%							
Test for overall effect: Z = 0.15 (P = 0.88)							
**Septic morbidity**							
Karakan 2007	1	15	2	15	3.1%	0.50 [0.05, 4.94]	2007
Plaudis 2012	2	30	2	28	4.6%	0.93 [0.14, 6.18]	2012
Wang 2013	8	62	13	61	25.4%	0.61 [0.27, 1.36]	2013
**Subtotal (95% CI)**		**107**		**104**	**33.2%**	**0.63 [0.31, 1.28]**	
Total events	11		17				
Heterogeneity: Tau² = 0.00; Chi² = 0.21, df = 2 (P = 0.90); I² = 0%							
Test for overall effect: Z = 1.28 (P = 0.20)							
**Total (95% CI)**		**497**		**469**	**100.0%**	**0.95 [0.63, 1.43]**	
Total events	43		43				
Heterogeneity: Tau² = 0.00; Chi² = 4.84, df = 7 (P = 0.68); I² = 0%							
Test for overall effect: Z = 0.23 (P = 0.82)							
Test for subgroup differences: Chi² = 0.67, df = 2 (P = 0.71), I² = 0%							

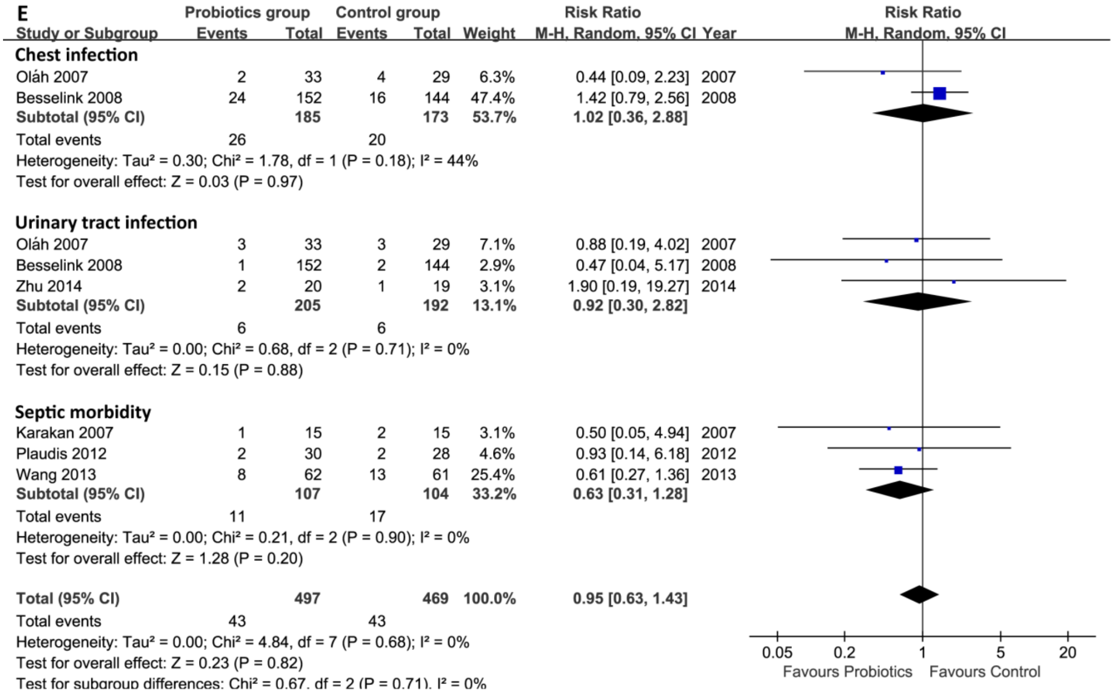

F

Study or Subgroup	Probiotics group Events	Probiotics group Total	Control group Events	Control group Total	Weight	Risk Ratio M-H, Random, 95% CI
Besselink 2008	75	152	76	144	100.0%	0.93 [0.75, 1.17]
**Total (95% CI)**		**152**		**144**	**100.0%**	**0.93 [0.75, 1.17]**
Total events	75		76			
Heterogeneity: Not applicable						
Test for overall effect: Z = 0.59 (P = 0.55)						

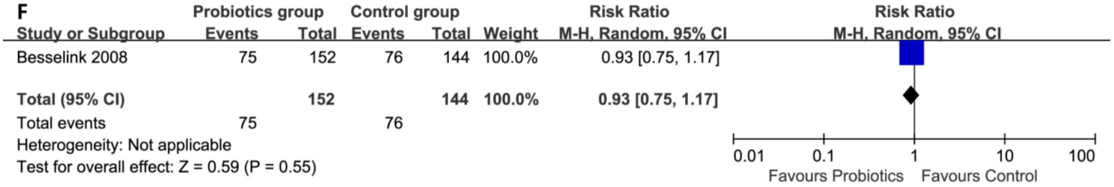

Figure S1. Meta-analysis on secondary outcomes. (A) surgical intervention, (B) systemic inflammatory response syndrome (SIRS), (C) multiple organ failure (MOF), (D) SIRS and MOF, (E) other infectious complications including chest infection, urinary tract infection and septic morbidity, and (F) use of antibiotics. CI, Confidence interval; M-H, Mantel-Haenszel.

Study or Subgroup	Probiotics group Events	Probiotics group Total	Control group Events	Control group Total	Weight	Risk Ratio M-H, Random, 95% CI	Year
**1.1.1 Probiotics alone**							
Besselink 2008	21	152	14	144	34.3%	1.42 [0.75, 2.69]	2008
Cui 2009	4	20	5	25	10.1%	1.00 [0.31, 3.24]	2009
Lata 2010	0	7	3	15	1.7%	0.29 [0.02, 4.89]	2010
Wang 2013	8	62	13	61	21.4%	0.61 [0.27, 1.36]	2013
Cui 2013	2	23	5	25	5.9%	0.43 [0.09, 2.03]	2013
Li 2014	5	27	8	28	14.4%	0.65 [0.24, 1.73]	2014
Zhu 2014	6	30	5	27	12.2%	1.08 [0.37, 3.14]	2014
**Subtotal (95% CI)**		**321**		**325**	**100.0%**	**0.90 [0.62, 1.30]**	
Total events	46		53				
Heterogeneity: Tau² = 0.00; Chi² = 4.96, df = 6 (P = 0.55); I² = 0%							
Test for overall effect: Z = 0.58 (P = 0.56)							
**1.1.2 Prebiotics alone**							
Karakan 2007	1	15	2	15	100.0%	0.50 [0.05, 4.94]	2007
**Subtotal (95% CI)**		**15**		**15**	**100.0%**	**0.50 [0.05, 4.94]**	
Total events	1		2				
Heterogeneity: Not applicable							
Test for overall effect: Z = 0.59 (P = 0.55)							
**1.1.3 Synbiotics alone**							
Oláh 2007	2	33	6	29	60.7%	0.29 [0.06, 1.34]	2007
Plaudis 2012	2	30	2	28	39.3%	0.93 [0.14, 6.18]	2012
**Subtotal (95% CI)**		**63**		**57**	**100.0%**	**0.46 [0.14, 1.51]**	
Total events	4		8				
Heterogeneity: Tau² = 0.00; Chi² = 0.88, df = 1 (P = 0.35); I² = 0%							
Test for overall effect: Z = 1.28 (P = 0.20)							
Test for subgroup differences: Chi² = 1.29, df = 2 (P = 0.53), I² = 0%							

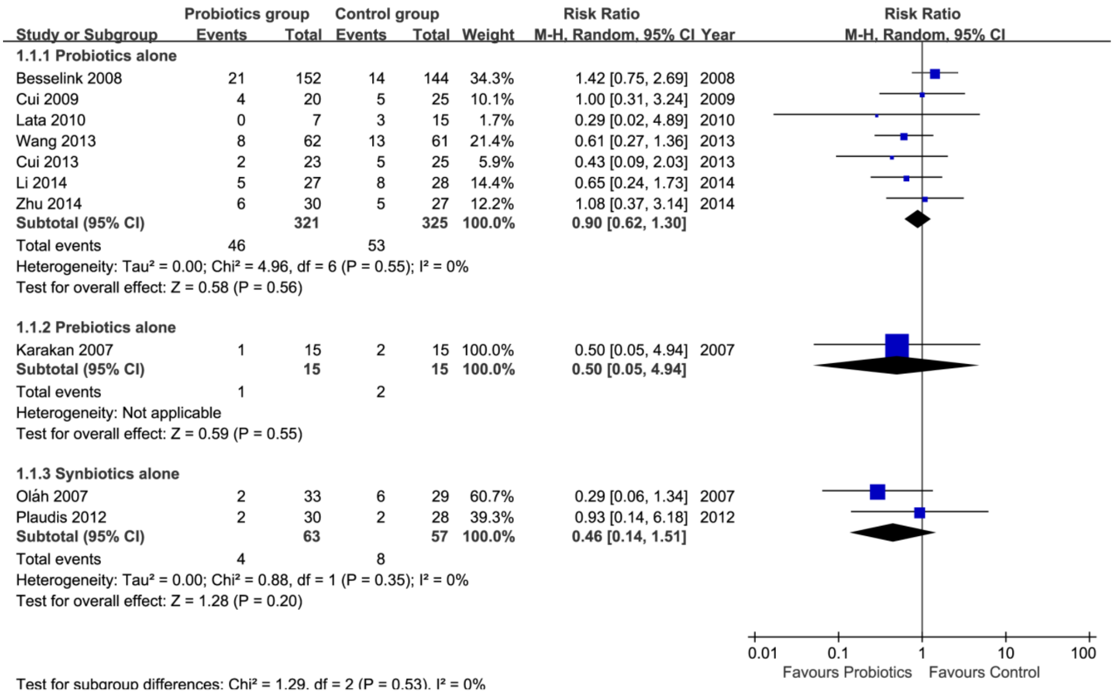

Figure 2A. Subgroup analysis of pre-, pro- and synbiotics alone in terms of infected pancreatic necrosis. M-H, Mantel-Haenszel; CI, confidence interval.

Study or Subgroup	Probiotics group Events	Probiotics group Total	Control group Events	Control group Total	Weight	Risk Ratio M-H, Random, 95% CI	Year
**1.3.1 Probiotics alone**							
Besselink 2008	24	152	9	144	29.2%	2.53 [1.22, 5.25]	2008
Cui 2009	1	20	2	25	6.1%	0.63 [0.06, 6.41]	2009
Wu 2009	5	14	6	13	23.7%	0.77 [0.31, 1.93]	2009
Lata 2010	0	7	0	15		Not estimable	2010
Cui 2013	1	23	1	25	4.6%	1.09 [0.07, 16.39]	2013
Wang 2013	5	62	6	61	18.5%	0.82 [0.26, 2.55]	2013
Li 2014	1	27	2	28	6.0%	0.52 [0.05, 5.39]	2014
Wu 2017	2	60	6	60	11.8%	0.33 [0.07, 1.59]	2017
**Subtotal (95% CI)**		**365**		**371**	**100.0%**	**0.98 [0.53, 1.81]**	
Total events	39		32				
Heterogeneity: Tau² = 0.20; Chi² = 8.67, df = 6 (P = 0.19); I² = 31%							
Test for overall effect: Z = 0.07 (P = 0.95)							
**1.3.2 Prebiotics alone**							
Karakan 2007	2	15	4	15	100.0%	0.50 [0.11, 2.33]	2007
**Subtotal (95% CI)**		**15**		**15**	**100.0%**	**0.50 [0.11, 2.33]**	
Total events	2		4				
Heterogeneity: Not applicable							
Test for overall effect: Z = 0.88 (P = 0.38)							
**1.3.3 Synbiotics alone**							
Oláh 2007	2	33	6	29	81.2%	0.29 [0.06, 1.34]	2007
Plaudis 2012	0	30	1	28	18.8%	0.31 [0.01, 7.35]	2012
**Subtotal (95% CI)**		**63**		**57**	**100.0%**	**0.30 [0.08, 1.17]**	
Total events	2		7				
Heterogeneity: Tau² = 0.00; Chi² = 0.00, df = 1 (P = 0.97); I² = 0%							
Test for overall effect: Z = 1.74 (P = 0.08)							
Test for subgroup differences: Chi² = 2.75, df = 2 (P = 0.25), I² = 27.4%							

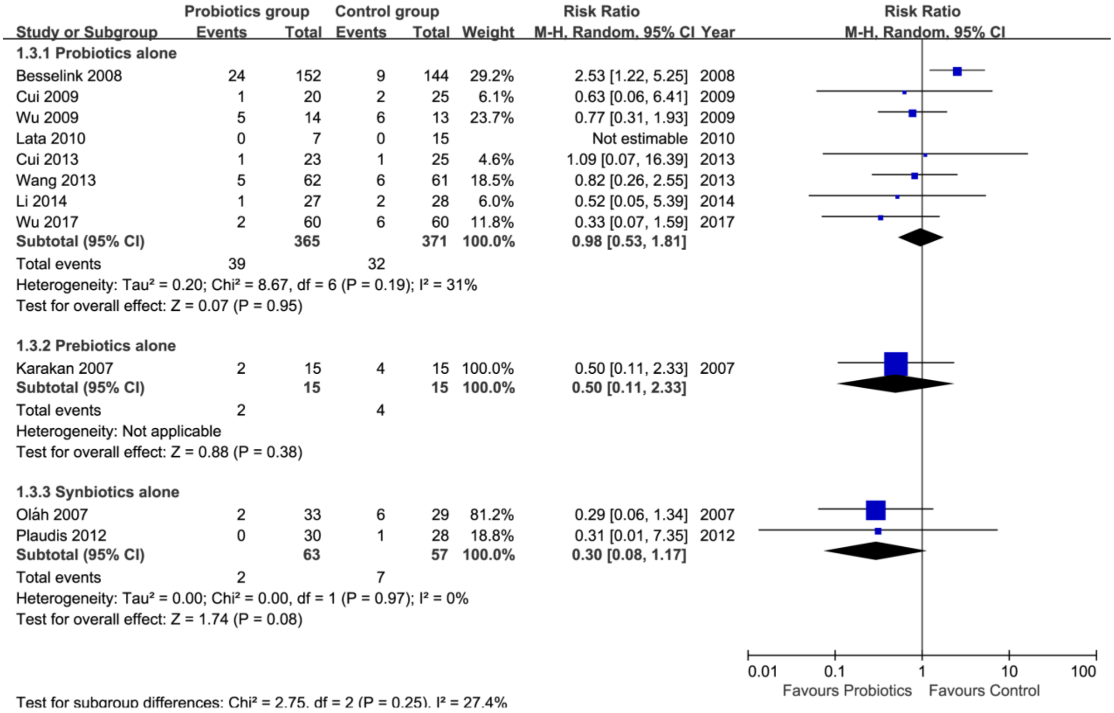

Figure 2B. Subgroup analysis of pre-, pro- and synbiotics alone with regard to mortality. M-H, Mantel-Haenszel; CI, confidence interval.

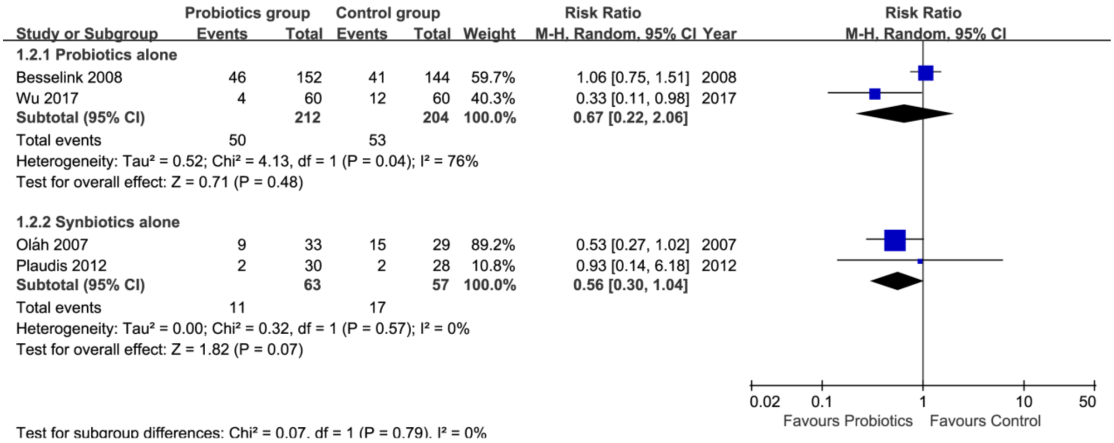

Figure 2C. Subgroup analysis of pre-, pro- and synbiotics alone with regard to total infection. M-H, Mantel-Haenszel; CI, confidence interval.

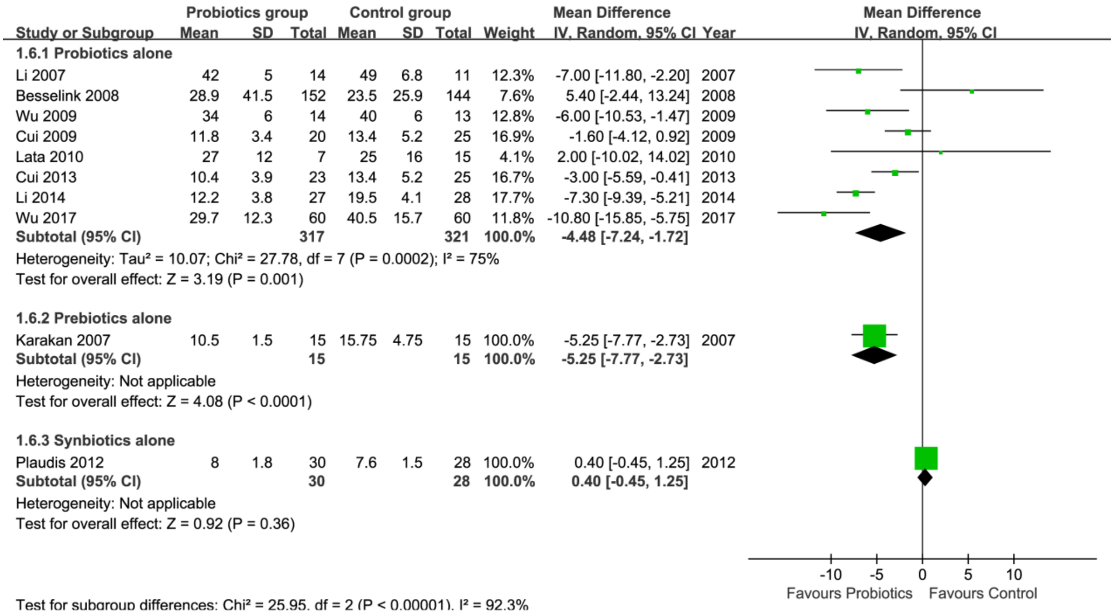

Figure 2D. Subgroup analysis of pre-, pro- and synbiotics alone with regard to length of hospital stay. M-H, Mantel-Haenszel; CI, confidence interval.

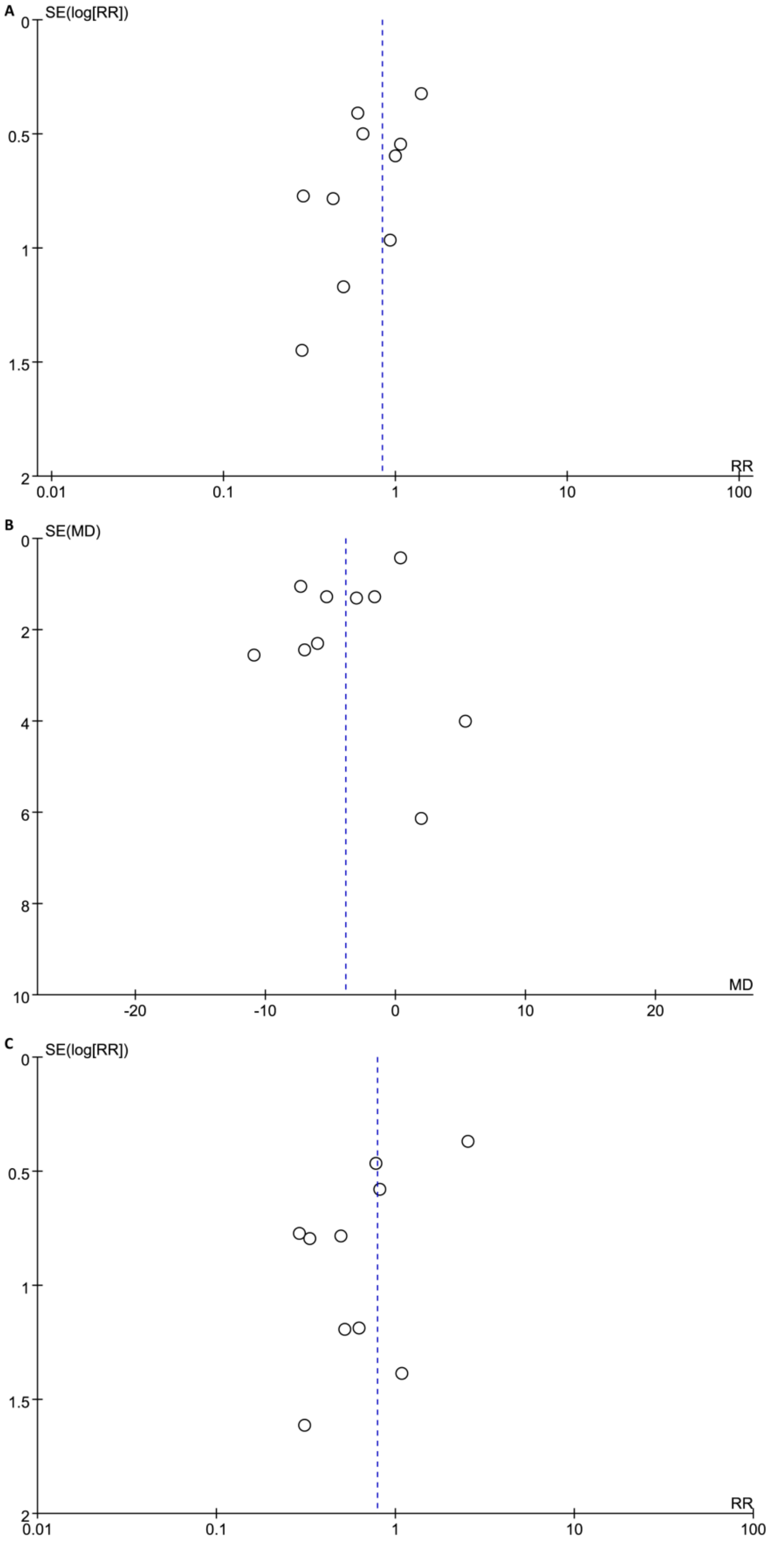

Figure S3. Funnel plot of (A) infected pancreatic necrosis, (B) mortality, and (C) length of hospital stay
